# Sensitivity of temporal heart rate variability in Poincaré plot to changes in parasympathetic nervous system activity

**DOI:** 10.1186/1475-925X-10-17

**Published:** 2011-03-03

**Authors:** Chandan K Karmakar, Ahsan H Khandoker, Andreas Voss, Marimuthu Palaniswami

**Affiliations:** 1 Department of Electrical and Electronic Engineering, The University of Melbourne, Parkville, VIC 3010, Australia; 2 Department of Medical Engineering & Biotechnology, University of Applied Sciences Jena, Germany

## Abstract

**Background:**

A novel descriptor (Complex Correlation Measure (CCM)) for measuring the variability in the temporal structure of Poincaré plot has been developed to characterize or distinguish between Poincaré plots with similar shapes.

**Methods:**

This study was designed to assess the changes in temporal structure of the Poincaré plot using *CCM *during atropine infusion, 70° head-up tilt and scopolamine administration in healthy human subjects. *CCM *quantifies the point-to-point variation of the signal rather than gross description of the Poincaré plot. The physiological relevance of *CCM *was demonstrated by comparing the changes in *CCM *values with autonomic perturbation during all phases of the experiment. The sensitivities of short term variability (*SD*1), long term variability (*SD*2) and variability in temporal structure (*CCM*) were analyzed by changing the temporal structure by shuffling the sequences of points of the Poincaré plot. Surrogate analysis was used to show *CCM *as a measure of changes in temporal structure rather than random noise and sensitivity of *CCM *with changes in parasympathetic activity.

**Results:**

*CCM *was found to be most sensitive to changes in temporal structure of the Poincaré plot as compared to *SD*1 and *SD*2. The values of all descriptors decreased with decrease in parasympathetic activity during atropine infusion and 70° head-up tilt phase. In contrast, values of all descriptors increased with increase in parasympathetic activity during scopolamine administration.

**Conclusions:**

The concordant reduction and enhancement in *CCM *values with parasympathetic activity indicates that the temporal variability of Poincaré plot is modulated by the parasympathetic activity which correlates with changes in *CCM *values. *CCM *is more sensitive than *SD*1 and *SD*2 to changes of parasympathetic activity.

## Background

Heart rate variability (HRV) is a powerful non-invasive method for analyzing the function of the autonomic nervous system. It is useful to understand the interplay between the sympathetic and parasympathetic autonomic nervous system, which serves to speed up and slow down the heart rate, respectively [1]. HRV, the variation of the time period between consecutive heart beats, is thought to reflect the heart's adaptability to the changing physiological conditions. It is dependent predominantly on the extrinsic regulation of the heart rate [2]. Assessment of HRV provides quantitative information about the modulation of heart rate (HR) by sympathetic nervous system (SNS) and parasympathetic nervous system (PNS). Interactions of SNS and PNS using HRV signal have been well studied and their importance established with a number of cardiac diseases including myocardial infarction [3], patients with congestive heart failure [4], patients at risk of sudden cardiac death [5,6] and patients with hypertension [7,8]. There are two main approaches to the analysis of HRV: time-domain and frequency-domain analysis. Time-domain indices (i.e., Mean, standard deviation (SD), standard deviation of normal RR intervals (SDNN), standard deviation of averaged normal RR intervals (SDANN) [9]) are derived from simple statistical calculations based on interbeat intervals (RR intervals). These indices are sensitive to transients and trends in the sample of heartbeats, and as such provide estimates of overall and beat-to-beat variability [10]. Frequency-domain analysis, which is based on the power spectral density of the heart rate time series, highlights the issue of the underlying rhythms of the mechanisms controlling heart rate (HR) and identified three major spectral peaks (high frequency (HF: 0.15-0.4 Hz), low frequency (LF: 0.04-0.15 Hz) and very low frequency (VLF: below 0.04 Hz)) in the adult HR spectrum [1]. These measurements can be derived from short-term (i.e 5 to 30 minutes) or long-term ECG recordings (i.e. 24 hours). HRV has been used as a non-invasive marker of the activity of the autonomic nervous system for over two decades. The necessary guidelines for comparing different studies of HRV have been established by the Task force of ESC and NPSE [9]. In [9], it has been suggested that the time-domain methods are ideal for the analysis of long-term HRV signal. Poincaré plot is one of the popular time domain HRV analysis techniques which is used both for short term (i.e. 5 to 30 minutes) or long term (ie. 24 hours) analysis.

Poincaré plot is a visual presentation of time series signal to recognize the hidden patterns. It is also a quantitative technique in the sense that it has various parameters (ex: short-term variability (*SD*1) and long-term variability (*SD*2)) to quantify the information from the plot. The Poincaré plot of HRV signal is constructed by plotting consecutive points of RR interval time series (i.e., lag-1 plot). It is a representation of HRV signal on phase space or Cartesian plane [11], which is commonly used to asses the dynamics of the HRV [12-15] signal, describe the sympathetic and parasympathetic modulation of heart rate [16,17] and in various clinical settings like diabetes [18], chronic heart failure [19], chronic renal failure [15] and sleep apnea syndrome [20].

**Figure 1:**
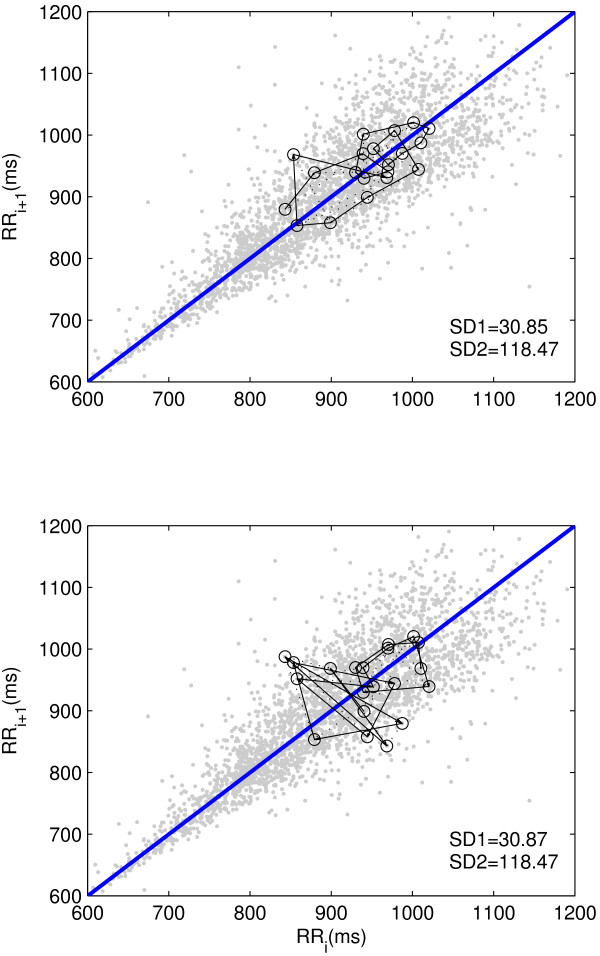
**Poincaré plots with similar *SD*1 and *SD*2 having different temporal dynamics**. Two different RR interval time series of length N (N = 2000) with similar *SD*1 and *SD*2 values having different temporal dynamics (first 20 points) are shown in top and bottom panel.

The popular technique used to quantify the Poincaré plot is fitting an ellipse to the shape of the Poincaré plot and measure the dispersion along the major and minor axis of the ellipse. This technique was first proposed by Tulppo et. al. [12] in which they have defined two standard descriptors of the plot *SD*1 and *SD*2 for quantification of the Poincaré plot geometry. Later, the description of *SD*1 and *SD*2 in terms of linear statistics, given by Brennan et. al. [21], showed that the standard descriptors guide the visual inspection of the distribution. In case of HRV, it reveals a useful visual pattern of the RR interval data by representing both short and long term variations of the signal [12,21]. The primary limitation of the standard descriptors used for quantifying Poincaré plot is the lack of embedding temporal information. The standard descriptors, *SD*1 and *SD*2, represent the distribution of signal in 2 D space and carries only information of width and length. As shown in Figure 1, Poincaré plots of similar *SD*1 and *SD*2 values can have completely different underlying temporal dynamics.

To overcome this limitation, in our previous study [22], we developed a novel measure, complex correlation measure (CCM), to quantify the temporal variation of the Poincaré plot. In that study, it was shown that *CCM *is more sensitive to changes in temporal structure of the signal than *SD*1 and *SD*2. In [22], it was reported that it is possible to have two Poincaré plots with similar *SD*1 and *SD*2 having varied temporal structure. In such a scenario, *CCM *can be used to successfully distinguish two Poincaré plots. *CCM *was also shown as a measurement from a series of lagged Poincaré plots (multiple lag correlation) which can potentially provide more information about the behavior of Poincaré plot than the conventional lag-1 plot measurements (*SD*1 and *SD*2). Moreover, *CCM *has shown to have better generalization capability over different pathology than *SD*1 and *SD*2, and it was reported as a novel parameter to characterize the variability in the temporal structure of the Poincaré plot.

Use of Poincaré plot of successive RR intervals to study the heart rate behavior during accentuated sympathovagal interaction has been reported in several studies [12,13,16]. In this study we demonstrate the physiological significance of the novel measure *CCM *by analyzing the effects of perturbations of autonomic function on Poincaré plot descriptors (*SD*1 and *SD*2) in young healthy subjects caused by the 70° head-up tilt test, atropine infusion and transdermal scopolamine patch. We also analyze the characteristics of the responses of *CCM *to changes in sequences of points in Poincaré plot by surrogate analysis, which provides insight into the variability in temporal structure of the Poincaré plot.

## Methods

### Complex Correlation Measure (*CCM*)

The *CCM *measures the point-to-point variation of the signal rather than gross description of the Poincaré plot. It is computed in a windowed manner which embeds the temporal information of the signal. A moving window of three consecutive points from the Poincaré plot are considered and the temporal variation of the points are measured. If three points are aligned on a line then the value of the variation is zero, which represents the linear alignment of the points. Moreover, since the individual measure involves three points of the two dimensional Poincaré plot, it is comprised of at least four different points of the time series for lag *m *= 1 and at most six points in case of lag *m *≥ 3. Hence the measure conveys information about four different lag correlations of the signal. If the Poincaré plot is composed of *N *points then the temporal variation of the plot, termed as *CCM *, is composed of all overlapping three point windows and can be calculated as:

(1) $$CCM(m)=\frac{1}{C_{n}(N-2)}\sum_{i=1}^{N-2}\|A(i)\|$$

where *m *represents lag of Poincaré plot, *A*(*i*) represents area of the *i-th *triangle and *C_n _*is the normalizing constant which is defined as, *C_n _*= π _* _*SD*1 _* _*SD*2, represents the area of the fitted ellipse over Poincaré plot at *lag-m*. The length of major and minor axis of the ellipse are 2*SD*1, 2*SD*2, where *SD*1, *SD*2 are the dispersion perpendicular to the line of identity (minor axis) and along the line of identity (major axis) respectively. The detail mathematical formulation of *CCM *is reported in our previous study [22].

### Sensitivity to changes in temporal structure

Literally the sensitivity is defined as the rate of change of the value due to the change in temporal structure of the signal. The change in temporal structure of the signal in a window is achieved by surrogating the signal (i.e, data points) in that window. In our previous study [22], we studied the temporal nature of sensitivity of *CCM *by changing the temporal structure of the signal using a moving fixed length window. In this study, the sensitivity of *CCM *was analyzed in order to define how it was affected by increasing amount of change in temporal structure. By increasing the number of surrogating points we have increased the probability of the amount of change in temporal structure of time-series signal. At each step the number of surrogated points is increased by 50, which gives enough resolution to understand the overall pattern. We calculated *SD*1, *SD*2 and *CCM *of a RR interval signal by increasing the number of surrogating points at a time. For a selected number of surrogating points, we have shuffled the points for 30 times and calculated all descriptors each time after shuffling. Finally the surrogated values of descriptors were taken as a mean of the calculated values. Finally, the sensitivity of descriptors Δ*SD*1*_j _*, Δ*SD*2*_j _*and Δ*CCM_j _*was calculated using equations 2-4:

(2) $$\Delta SD1_{j}=\frac{SD1_{j}-SD1_{0}}{SD1_{0}}$$

(3) $$\Delta SD2_{j}=\frac{SD2_{j}-SD2_{0}}{SD2_{0}}$$

(4) $$\Delta CCM_{j}=\frac{CCM_{j}-CCM_{0}}{CCM_{0}}$$

where *SD*1_0_, *SD*2_0 _and *CCM*_0 _were the parameters measured for the original data set without surrogation and j represents the window number whose data was surrogated. Moreover, *SD*1*_j_*, *SD*2*_j _*and *CCM_j _*represent the *SD*1, *SD*2 and *CCM *values respectively after surrogation of *j^th ^*step.

### Surrogate analysis

To show statistically that *CCM *is a measure of temporal variation rather than the outcome of a random process with no temporal variations, we adapted one method of surrogate data introduced by Theiler et al. [23]. We have used this method to prove, the hypothesis that the correlation properties of RR interval were distinguishable from uncorrelated random noise. This also indicates that the effect of surrogation is higher in case of a strongly correlated signal. In this study, 30 surrogate RR interval series were generated for each RR interval time series by shuffling the original RR interval time series. Each of the surrogated RR interval series had the identical statistical distribution (mean, SD, higher moments) as surrogation differed only in the temporal sequence from the original time series. The effect of surrogation on the Poincaré plot was then measured by calculating *SD*1, *SD*2 and *CCM *for each surrogated time series and the means of the surrogated values (*MeanSD*1*_s _*, *MeanSD*2*_s _*and *MeanCCM_s_*) were then calculated for 30 surrogated time series and compared to the *SD*1*_i _*, *SD*2*_i _*and *CCM_i _*of original time series to determine the sensitivity of all parameters. The test of the hypothesis was performed by computing the relative changes of *SD*1, *SD*2 and *CCM *values (*SD*1*_R _*, *SD*2*_R _*and *CCM_R _*respectively) between original and the mean of surrogated time series. That is, for *i*th time series it was defined as:

(5) $$SD1_{R}=\frac{\|(SD1_{i}-MeanSD1_{s})\|}{SD1_{i}}$$

(6) $$SD2_{R}=\frac{\|(SD2_{i}-MeanSD2_{s})\|}{SD2_{i}}$$

(7) $$CCM_{R}=\frac{\|(CCM_{i}-MeanCCM_{s})\|}{CCM_{i}}$$

### Subjects and Study design

In this study, five subjects with normal sinus rhythm, did not smoke, had no cardiovascular abnormalities and were not taking any medications were studied. Subjects were aged between 20 and 40 years (30.2 ± 7.2 year). All subjects had provided fully informed consent and ethical approval was granted by the Austin Hospital Committee of Ethics in Human Research.

All studies were performed at the same time of the day without any disturbances. No respiration control was performed because all phases of the study were conducted in the resting state. An intravenous cannula was inserted into an antecubital vein and subjects then rested for 20 minutes before commencement of data collection. The length of the study varies from 10 to 20 minutes. For autonomic perturbations the following sequence of protocol was performed. At least 20 minutes was allowed between each phase to permit the heart rate to return baseline. The sequence of phases was maintained strictly as follows:

#### Baseline study

All baseline studies were conducted in subjects in the post-absorptive state after resting for 10 minutes in the supine position.

#### Seventy degree head-up tilt

Data were collected after subjects were tilted 70° on a motorized table. This maneuver increases sympathetic and decreases parasympathetic nervous system activity [24]. To permit the heart rate to stabilize at the new position, data were collected 5 minutes after the subjects were tilted.

#### Atropine infusion

Atropine sulphate (1.2 mg) was added to 50 ml of intravenous dextrose and infused at a rate of 0.12 mg/min for 5 minutes and then at a rate of 0.24 mg/min until completion of this phase of study. Use of this dose regimen reduces parasympathetic nervous system activity significantly [25]. After 10 minutes of infusion of atropine, the data collection started.

#### Transdermal scopolamine

One week after the above studies, a low-dose transdermal scopolamine patch (hyoscine 1.5 mg) was applied overnight to an undamaged hair free area of skin behind the ear. The patch remained in situ for the duration of this period of the study. It has been shown in [26] that low-dose transermal scopolamine increases parasympathetic nervous system activity.

Details of the study design and data collection were published in [16].

## Results

**Figure 2:**
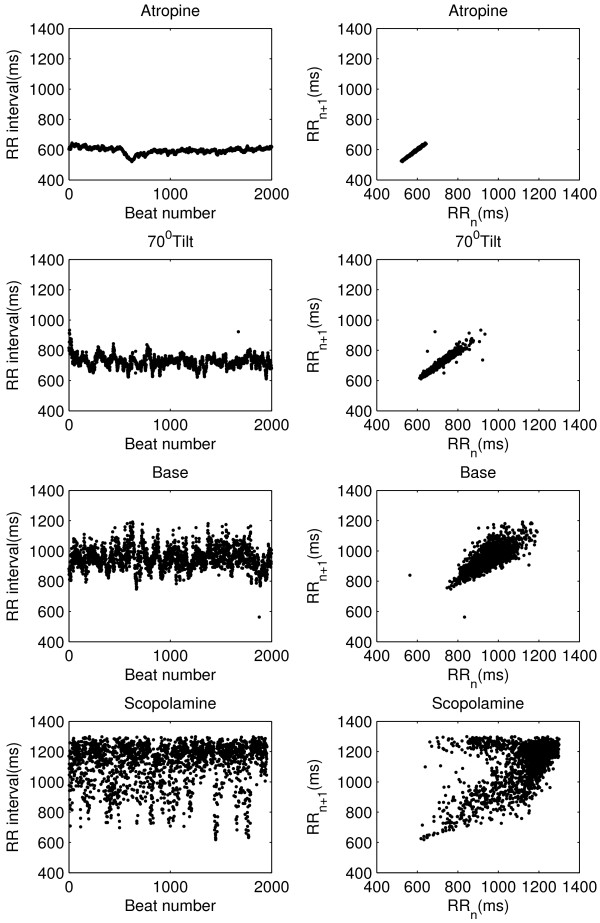
**RR intervals and Poincaré plots during autonomic perturbations**. RR interval time series for single subject from all four phases of study with corresponding Poincaré plot.

In Figure 2, the RR intervals and the corresponding Poincaré plot for all four phases of the experiment with the same subject are shown. From figure it is eminent that the atropine infusion strongly reduces the size of plot by reducing both the RR interval (increase in heart rate) and its variation. Whereas, the head-up tilt position reduces the RR interval (increase in heart rate) variability markedly with respect to the baseline. In contrast, use of low-dose transdermal scopolamine increases the RR interval (reduces heart rate) and its variability resulted into a wider Poincaré plot in terms of width in both direction (perpendicular to line of identity and along the line of identity).

Table 1 summarizes the mean and standard deviation of heart rate variability features of all subjects in all four phases. Short-term variability (*SD*1) was increased in Scopolamine phase and decreased in Atropine phase. A similar trend was also found for long-term variability (*SD*2). Changes of *SD*1 values from phase to phase was much higher than that of *SD*2. *CCM *value was also minimum in Atropine phase and maximum at Scopolamine phase. Changes in mean values of *CCM *between study phases were higher than both *SD*1 and *SD*2 (Table 1). Moreover, change in *CCM *values in Atropine, 70° head-up tilt and Scopolamine phases from Baseline are found significant (*p *< 0.01). *SD*1 values were significantly different in Atropine and Scopolamine phases and *SD*2 values differ only in Atropine phase.

**Table 1:** *MEAN *and Standard deviation *SD *of values of all descriptors for *lag-1 *Poincaré plot

Feature	SD1	SD2	CCM
	(mean ± sd) (ms)	(mean ± sd) (ms)	(mean ± sd)
Atropine	4.45 ± 2.45*	43.11 ± 13.79*	3.88E-02 ± 1.05*E *− 02*
Head-up tilt	11.96 ± 5.47	70.77 ± 13.98	6.29E-02 ± 2.08*E *− 02*
Baseline	28.74 ± 9.28	85.94 ± 11.27	1.50E-01 ± 3.40E-02
Scopolamine	69.90 ± 21.25*	103.05 ± 20.05	2.75E-01 ± 2.14*E *− 02*

*SD*1, *SD*2 and *CCM *values of all subjects (N = 5) were calculated for four phases as described in section.

* indicates the value of the feature in corresponding phase is significantly (*p *< 0.01) different from baseline phase using Wilcoxon rank sum test.

**Figure 3:**
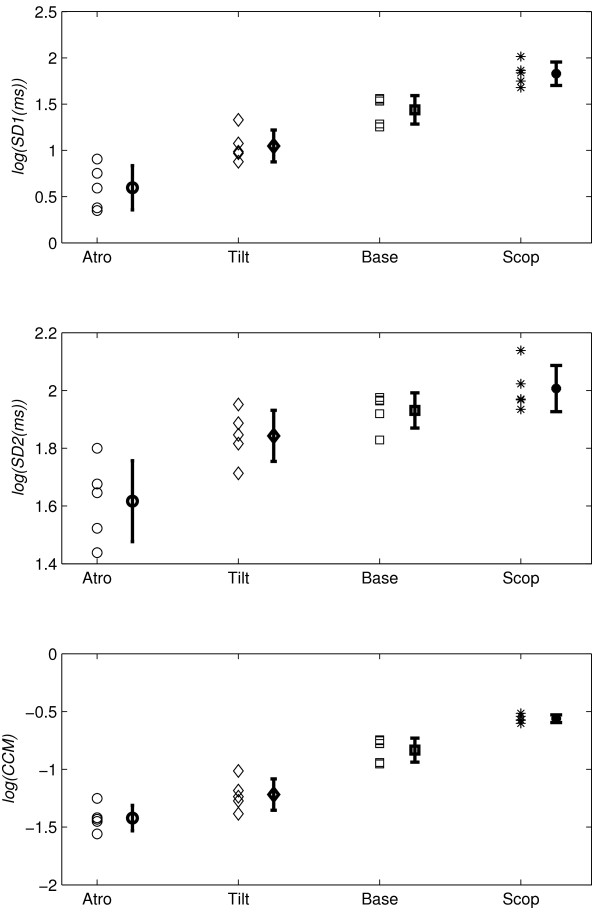
**Errorbars of *SD*1, *SD*2 and *CCM***. Errorbar (n = 5) of *log*(*SD*1), *log*(*SD*2) and *log*(*CCM*) for Atropine, 70° head-up tilt, baseline and scopolamine phase. All values were calculated for short segment (~ 20 minutes) RR interval time series signal.

The errorbars of log-scaled *SD*1, *SD*2 and *CCM *values for four groups of subjects are shown in Figure 3. The atropine administration resulted into reduction in mean value of *SD*1 (*SD *of Δ*RR*) for all subjects. The similar effect was also found for *SD*2 and *CCM *. The use of scopolamine patch increased both the width and height of the Poincaré plot which resulted into increase in mean values of *CCM *as well as *SD*1, *SD*2. All subjects have shown a marked reduction in *SD*1, *SD*2 and *CCM *values in 70° head-up tilt phase compared to the baseline.

**Figure 4:**
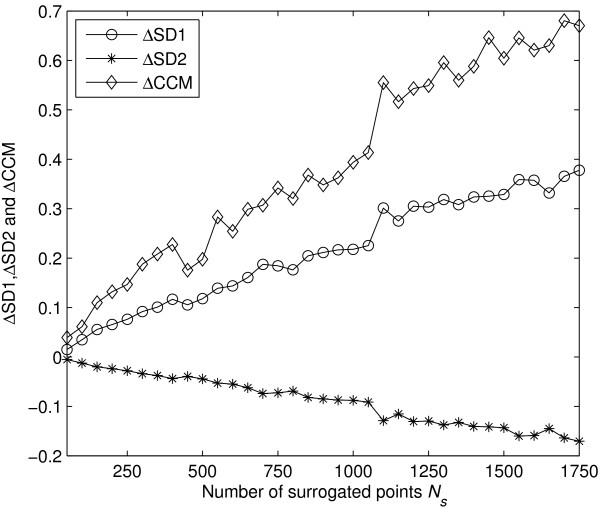
**Sensitivity of *SD*1, *SD*2 and *CCM *with changes in temporal dynamics**. Sensitivity of *SD*1, *SD*2 and *CCM *with number of shuffled points *N_s_*. At each step the number of shuffled points increased by 50. Each time the signal has been shuffled for 30 times and its mean has been taken to calculated the sensitivity.

Figure 4 represented the change of descriptors *SD*1, *SD*2 and *CCM *with increasing number of shuffled RR intervals. From Figure 4 it is obvious that the rate of change with number of shuffled RR intervals was highest for *CCM *at any point than *SD*1 and *SD*2.

**Figure 5:**
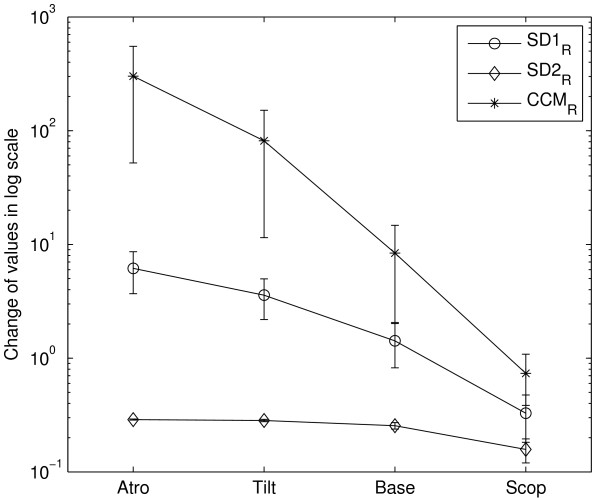
**Changes in *SD*1, *SD*2 and *CCM *values for all phases**. *SD*1*_R _*, *SD*2*_R _*and *CCM_R _*is the change between original and surrogated values of *SD*1, *SD*2 and *CCM *(given by number of original *Mean*). RR interval signal of each subject for four different phases was randomly shuffled 30 times then *SD*1, *SD*2 and *CCM *was calculated each time. *Mean *of each descriptor (*SD*1, *SD*2 and *CCM*) of these 30 data sets was compared to value of corresponding descriptor (*SD*1, *SD*2 and *CCM*) of originally ordered RR interval signal.

Surrogate data testing was performed to test if *CCM *can represent a measure of temporal dynamics of RR intervals and quantify sensitivity of those parameters to relative changes. For each subject at all four phases, relative changes in *SD*1, *SD*2 and *CCM *values of the RR interval signal were calculated. Figure 5 shows the log scaled values of relative changes in *SD*1, *SD*2 and *CCM *at all four phases of the study using SD errorbar. Relative changes in *CCM *values were higher compared to relative changes in *SD*1 and *SD*2 values due to shuffling the sequences of data series (Figure 5). Moreover, the relative changes were found to be lowest for *SD*2 for all four phases.

## Discussion

Heart rate variability time series were analyzed using a variety of linear methods, most commonly using HRV descriptive statistics in the time and frequency domains [1,10,12,14,16]. The potential use of Poincaré plot as a serial correlation technique has also been explored to quantify autonomic activity [16,27]. In this study, we have shown that *CCM *(a measure of temporal dynamics) for Poincaré plot provides a dynamical way to quantify autonomic activity. In addition to this, *CCM *has been shown to be a more sensitive parameter compared to *SD*1 and *SD*2 to any changes of dynamics in autonomic activity.

### Physiological relevance of CCM

Quantitative Poincaré plot analysis was used to assess the changes in HRV during parasympathetic blockade [12] and compared the results with power spectral analysis of HRV, which was the commonly used method in the measurement of sympathovagal interaction [1,12,28,29]. It was also reported that Poincaré analysis method can provide the heart rate dynamics that is not detected by the conventional time domain methods [12]. The present quantitative analysis was performed to measure the instantaneous beat-to-beat variance of RR intervals (*SD*1), the long term continuous variance of all RR intervals (*SD*2) and the variation in temporal structure of all RR intervals (*CCM*). Instantaneous changes in RR intervals are mediated by vagal efferent activity, because vagal effects on the sinus node are known to develop faster than sympathetically mediated effects [30,31]. The maximum reduction in *SD*1 during atropine infusion compared to baseline values, confirming that *SD*1 quantifies the vagal modulation of heart rate, which was also reported by Kamen et. al. [16] and Tulppo et. al. [12]. Similar reduction in *CCM *value could be observed (Table 1 and Figure 3), which indicates that *CCM *might correlate the parasympathetic nervous system activity. The lowest value of *CCM *has also been found during atropine infusion which reduced the parasympathetic activity and reduces instantaneous changes in HRV signal. Moreover, significant (*p *< 0.01) change in *CCM *values in all phases from Baseline phase compared to *SD*1 and *SD*2 indicates that *CCM *is more sensitive to changes in parasympathetic activity (Table 1). On the contrary, changes in *SD*1 values are insignificant in 70° Head-up tilt phase and changes in *SD*2 values are insignificant both in 70° Head-up tilt as well as Scopolamine phases.

Reciprocal changes in sympathetic and parasympathetic activity occurs during head-up tilt phase. The RR interval and the high-frequency power of RR intervals decreases during the head up tilt phase as evidence of withdrawal of vagal activity (decrease in parasympathetic activity) [32-34]. The short term variability measure of Poincaré plot (*SD*1) also decreases and correlates with high-frequency power as reported by Kamen et. al. [16]. In this study, *SD*1 value decreased during 70° head-up tilt phase compared to baseline, which supports the results reported by previous studies [16,24]. The *CCM *value has also decreased in 70° head-up tilt phase compared to baseline, which indicates that *CCM *value is modulated by the vagal tone (parasympathetic activity). Therefore, changes in autonomic regulation caused by 70° head-up tilt phase resulted in concordant changes in the temporal structure of the Poincaré plot of RR intervals.

The low-dose transdermal scopolamine patch (hyoscine 1.5 mg) may decrease heart rate by a paradoxical vagomimetic effect [26]. Delivery by transdermal patch substantially increases both baseline and reflexly augmented levels of cardiac parasympathetic activity over 24 hours in normal subjects [35,36]. Both time-domain HRV (Mean, SD) and frequency domain HRV (high frequency power) increased to a greater extent during administration of low-dose scopolamine, which indicates the increase in parasympathetic activity [26]. The increase in parasympathetic activity decreases the heart rate and increases the RR interval as well as instantaneous variance in the RR, as measured by *SD*1 of Poincaré plot. The increased value of SD1 correlates with increased high frequency power and is supported by the previous study reported by Kamen et. al. [16]. In this study, the variability in the temporal structure of the Poincaré plot (measured as *CCM*) was also found to be increased with increase in parasympathetic activity during administration of low-dose scopolamine (Figure 3, Table 1). The increase in *CCM *value indicates that it reflects the change in parasympathetic activity harmoniously.

### Sensitivity of CCM due to changes in dynamics

In this study, we have found that *CCM *correlates with the parasympathetic activity similar to *SD*1 [16]. In [22], we have shown that *CCM *is sensitive to change in temporal structure of the signal irrespective of temporal position of the signal. In that study, we had used simulated RR interval signal to prove our hypothesis. In line with the previous finding [22], in this study the relation of *CCM *with increasing number of shuffled RR intervals has been studied. The highest rate of change of *CCM *with number of shuffled RR intervals (Figure 4) at any point represents the maximum sensitivity of *CCM *with respect to change in temporal structure of the Poincaré plot. Therefore, we can conclude that *CCM *is much more sensitive than *SD*1 and *SD*2 with respect to change in temporal structure or the change in autocorrelation of the signal which was earlier reported in [22]. Moreover, sensitivity of *CCM *with small number of RR intervals increases its applicability to short length HRV signal analysis. However, it is not possible to determine the value of minimum number of required RR interval for all biomedical application as it will be problem specific rather than a global one.

The impact of changes in parasympathetic activity on temporal structure of the Poincaré plot is obvious from Figure 5. The changes due to surrogating are the highest for *CCM *in all phases, which might indicate that *CCM *is a measure of temporal structure of the plot and more sensitive to it than *SD*1 and *SD*2. Moreover, the change in its value between before and after surrogating is the highest for atropine phase which might indicate the reducing parasympathetic activity and its impact on the temporal structure of the plot better manifest in *CCM *value. In atropine phase, since the parasympathetic activity is reduced, variability decreases (low *SD*1 values) which is reflected by substantially linear temporal structure of the plot (lower *CCM *values). After surrogating, the correlation among the signal vanishes and as a result, uncorrelated or random temporal structure increased the *CCM *value. Therefore, the difference between original and surrogate value indicates that *CCM *depends on the correlation properties of the RR interval and it can be used to distinguish the HRV signal from uncorrelated random noise. Moreover, the difference between original and surrogate value also indicates the sensitivity of the *CCM *increases with degree of blocking parasympathetic activity by 70° head-up tilt and atropine infusion. On the other hand, the sensitivity of *CCM *decreases with enhancement of parasympathetic activity by scopolamine administration.

## Conclusion

By using the quantitative Poincaré plot analysis of HRV signal, we observed that atropine infusion, 70° head-up tilt and scopolamine administration result in changes in heart rate variability [short term variation (*SD*1) as well as long term variation (*SD*2)] and heart rate dynamics [temporal structure (*CCM*) values]. Subtle differences in dynamics of HRV signal were detected by *CCM *in all phases of the study. These observations provide some novel information on the physiological relevance of *CCM *for Poincaré plot analysis: 1) The variability of temporal structure of Poincaré plot of HRV, quantified using *CCM*, correlates the parasympathetic activity 2) *CCM *is highly sensitive to changes in parasympathetic activity (vagal tone) as compared to *SD*1 and *SD*2. Although *CCM *captures temporal variation of Poincaré plot, it fails if the RR intervals are aligned on a line. However, existence of few zero area patterns does not affect the overall *CCM *value as it is measured using a moving window of three consecutive points. Further studies of *CCM *of HRV signal with changes in sympathetic activity may give the complete physiological explanation of *CCM *with respect to sympathovagal activity. Moreover, due to well published changes in autonomic regulation between men and women and in different age groups [37], and investigation of gender and age effects on *CCM *would be of interest in further studies.

## Competing interests

The authors declare that they have no competing interests.

## Authors' contributions

CKK conceived, derived and implemented the new descriptor, generated experimental results data and drafted the manuscript with supervision of AHK and MP. AHK, AV and MP contributed to the development of the new descriptor and participated in the discussion and interpretation of the results. All authors read and approved the final manuscript.
